# From Isostructurality to Structural Diversity of Ag(I) Coordination Complexes Formed with Imidazole Based Bipodal Ligands, and Disclosure of a Unique Topology

**DOI:** 10.3390/ma15051852

**Published:** 2022-03-01

**Authors:** Liliana Dobrzańska

**Affiliations:** Faculty of Chemistry, Nicolaus Copernicus University in Toruń, Gagarina 7, 87-100 Toruń, Poland; lianger@umk.pl

**Keywords:** SCXRD, coordination polymers, topology, intermolecular interactions

## Abstract

Two Ag(I) complexes with 1,3-bis(imidazol-1-ylmethyl)benzene (**bib**) and counterions BF_4_^−^ (**1**) and PF_6_^−^ (**2**) were synthesized in order to check their behavior in forming molecular/crystal structures. This allows comparison with the final products of analogous syntheses performed with similar bidentate ligands containing methyl substituents on the benzene ring, namely 1,3-bis(imidazol-1-ylmethyl)-5-methylbenzene (**bimb**) and 1,3-bis(imidazol-1-ylmethyl)-2,4,6-trimethylbenzene (**bitmb**). The Ag(I) complexes obtained with the methylated ligands mentioned above form isostructural pairs of waved 1D chains or dinuclear boxes, of general formula {[Ag(**bimb**)]X}_n_ and [Ag_2_(**btmb**)_2_]X_2_, respectively (X = BF_4_^−^, PF_6_^−^), under the same reaction conditions. SCXRD analyses of **1** and **2** revealed the formation of polymeric coordination compounds of formula {[Ag_2_(**bib**)_3_](BF_4_)_2_}_n_ and {[Ag(**bib**)]PF_6_}_n_, respectively, different from those observed for **bimb**. The 3D coordination polymer **1** forms a unique 5,5-c net of 5,5T188 topological type, observed for the very first time for a coordination compound, with silver cations adopting a trigonal geometry, whereas **2** shows the presence of 1D single-stranded cationic helices with linear coordination of the metal centers. Interestingly, these complexes differ not only from the mentioned isostructural pairs of related Ag(I) complexes, but also from the isostructural pair of compounds obtained as the final product when reacting **bib** and **bimb** with the larger counterion CF_3_SO_3_^−^. Hirshfeld surface analyses indicate a higher contribution of F⋯H intermolecular contacts in **2** than in **1**, with H⋯H contacts being dominant in the latter.

## 1. Introduction

**Figure 1:**
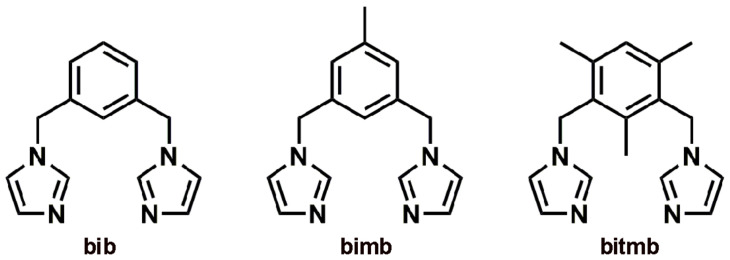
Schematic representation of the ligands: 1,3-bis(imidazol-1-ylmethyl)benzene (**bib**), 1,3-bis(imidazol-1-ylmethyl)-5-methylbenzene (**bimb**) and 1,3-bis(imidazol-1-ylmethyl)-2,4,6-trimethylbenzene (**bitmb**).

Controlling the formation of crystal structures is still far out of reach. Even minor modifications of the molecular composition can lead to enormous differences in the crystalline products. This is not so surprising considering that crystal structure prediction methods can generate hundreds of forms which show little difference in total lattice energy, even for simple organic molecules of the same composition [1,2]. Obviously, molecular flexibility adds more complexity to this matter. Systematic studies on isostructurality (equivalence of crystal structures) [3,4], as well as the occurrence of polymorphism (multiple crystalline forms of particular composition) [5,6] allow us to gain some insight into this topic. Recently, we have shown that 1,3-bis(imidazol-1-ylmethyl)benzene (**bib**) and 1,3-bis(imidazol-1-ylmethyl)-5-methylbenzene (**bimb**) form isostructural 1D Ag(I) complexes with CF_3_SO_3_^−^ as counterion, of general formula {[Ag**L**]CF_3_SO_3_}_n_ [L = **bib** or **bimb,** Figure 1], under the same reaction conditions [7]. Reacting **bimb** with silver salts containing counterions of a smaller molecular volume and different spherical shape, such as BF_4_^−^ and PF_6_^−^, leads to the formation of isostructural polymeric (1D) compounds, which are not isostructural with those formed by CF_3_SO_3_^−^, despite having a similar composition [8]. Performing the reaction with AgBF_4_ and AgPF_6_ and a ligand containing three methyl substituents, namely 1,3-bis(imidazol-1-ylmethyl)-2,4,6-trimethylbenzene (**bitmb**), leads to the formation of an isostructural pair of discrete molecular boxes of general formula [Ag_2_(**bitmb**)_2_]X_2_ where X = BF_4_^−^ or PF_6_^−^, under the same conditions [9,10]. In continuation of our studies on preferential crystal structure formation by compounds based on dipodal imidazole based ligands [7,8,9,10,11], Ag(I) complexes with **bib** and counterions BF_4_^−^ and PF_6_^−^ were synthesized. Interestingly, the final products are not isostructural with one another, and neither with the 1D polymers formed with CF_3_SO_3_^−^ (refcodes: WAJGUN (**bib**), WAJHAU (**bimb**)). They also differ from the Ag(I) complexes obtained with **bimb** or **bitmb** under the same reaction conditions (refcodes: not available yet (**bimb**); NUYQIJ and TAPLUT (**bitmb**)). Moreover, using BF_4_^−^ as a counterion led to a unique topology, which had not been observed before for coordination polymers.

## 2. Materials and Methods

### 2.1. Reagents and Materials

All commercially available chemicals were of reagent grade and were used without further purification. The ligand 1,3-bis(imidazol-1-ylmethyl)benzene (**bib**) was synthesized by the S_N_2 reaction of imidazole with 1,3-bis(bromomethyl)benzene in MeOH (white solid, 38% yield), as reported earlier [7]. Anal. calc. for C_14_H_14_N_4_·2H_2_O: C, 61.3; H, 6.6; N, 20.4. Found: C, 60.9; H, 6.7; N, 20.3%. ^1^H NMR (CDCl_3_, 600 MHz) δ 7.53 (s, 2H), 7.35 (t, J = 7.7 Hz, 1H), 7.11–7.08 (m, 4H), 6.92 (br s, 1H), 6.88 (s, 2H), 5.10 (s, 4H); 13C NMR (CDCl3, 150 MHz) δ 137.4, 137.2, 130.0, 129.7, 127.1, 125.9, 119.2, 50.4.

### 2.2. Measurements

^1^H and ^13^C NMR spectra were recorded on a Bruker Avance 600 MHz instrument and referenced to residual solvent peaks. IR spectra were recorded with a PerkinElmer 2000 FT-IR spectrometer. Thermal analysis studies for **1** and **2** were performed on a TA Instruments SDT 650 analyzer at a heating rate of 2 °C min^−1^ under dry nitrogen with a flow rate of 100 mL min^−1^.

### 2.3. Synthesis of Ag(I) Complexes

The syntheses of {[Ag_2_(**bib**)_3_](BF_4_)_2_}_n_ (**1**) and {[Ag(**bib**)]PF_6_}_n_ (**2**) were performed in a dark environment. A solution of a particular silver salt, such as silver tetrafluoroborate or silver hexafluorophosphate (0.1 mmol), in acetonitrile (10 mL) was added to a solution of 1,3-bis(imidazol-1-ylmethyl)benzene (0.1 mmol) in acetonitrile (30 mL). The mixture was stirred for a few minutes and then left to undergo slow evaporation. After 3–4 weeks, colorless crystals were obtained. IR (cm^−1^): (**1**) 3131 (w), 1591 (w), 1512 (m), 1447 (w), 1401 (w), 1348 (w), 1237 (m), 1047 (s), 926 (m), 812 (m), 727 (vs); (**2**) 3139 (w), 1610 (w), 1522 (m), 1450 (w), 1362 (w), 1242 (m), 1093 (s), 1029 (w), 953 (w), 817 (vs), 727 (vs).

### 2.4. Structure Determination

Single crystal X-ray diffraction data for **1** and **2** were collected on a Bruker APEX2 diffractometer [12] equipped with graphite monochromated MoKα radiation (λ = 0.71073Å). The crystals were mounted on a glass fiber and coated with Paratone-N oil. Data collection was carried out at 100(2) K to minimize solvent loss, possible structural disorder and thermal motion effects. Cell refinement and data reduction were performed using the program SAINT [13] and all empirical absorption corrections were performed using SADABS [14]. The structures were solved by using direct methods with SHELXS-2018/3 [15] and refined by using full-matrix least-squares methods based on *F*^2^ by using SHELXL-2018/3 [16]. The programs Mercury [17] and POV-Ray [18] were both used to prepare molecular graphics. All non-hydrogen atoms were refined anisotropically, and the hydrogen atoms were positioned geometrically with C-H = 0.95 Å (aromatic) and 0.99 Å (methylene) and refined as riding, with *U*iso(H) = 1.2 *U*eq (C). A summary of the data collection and structure refinement parameters is provided in Table 1. The crystallographic data for compounds **1** and **2** have been deposited at the Cambridge Crystallographic Data Centre: CCDC 2,105,343 for **1** and CCDC 2,105,344 for **2**. These data can be obtained free of charge from The Cambridge Crystallographic Data Centre via http://www.ccdc.cam.ac.uk/structures (accessed on 28 December 2021).

## 3. Results and Discussion

So far, three crystal structures of Ag(I) complexes with **bib** (not taking into account structures with mixed ligands) have been deposited in the CSD (ConQuest Version 2021.2.0), with counterions such as CF_3_SO_3_^−^ (**A**) [7] CN^−^ (**B**, refcode: PEVBAU) and SCN^−^ (**C**, refcode: PEVBIC) [19]. The last two complexes were obtained upon reaction with excess of metal salt (ca. 2:1 M:L ratio), but only one of these metal complexes, namely that with SCN^−^, reflects this excess, as the coordination compound with CN^−^ shows a 1:1 M:L molar ratio. Both complexes form 2D coordination polymers. The silver ions show a trigonal or distorted tetrahedral coordination in **B** and **C**, respectively. Using triflate as the counterion with molar ratio 1 to 1 (M:L) leads to the formation of 1D waved chains with the silver ions showing a linear geometry. The latter compound is isostructural with the corresponding compound obtained with **bimb** under similar conditions. The Ag(I) complexes of the latter ligand with BF_4_^−^ and PF_6_^−^ are isostructural and form 1D chains. However, as a result of the smaller size and different shape of these counterions, the silver complexes are not isostructural with those formed with triflate. 

### 3.1. Crystal Structure of the Ag(I) Complex with BF_4_^−^ as Counterion ({[Ag_2_(**bib**)_3_](BF_4_)_2_}_n,_ **1**)

**Figure 2:**
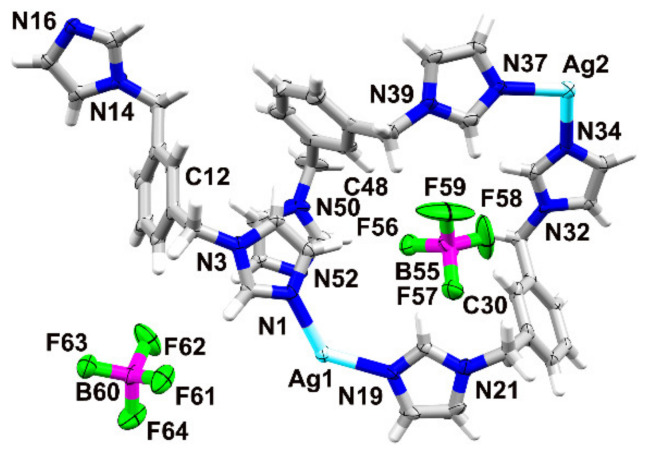
Asymmetric unit of **1**, atomic displacement plot is shown at 50% probability; selected labels presented.

The complex crystallizes in the monoclinic space group *Cc*, with three crystallographically independent ligand molecules adopting an *anti*-conformation, two metal ions and two counterions in the asymmetric unit (Figure 2).

**Figure 3:**
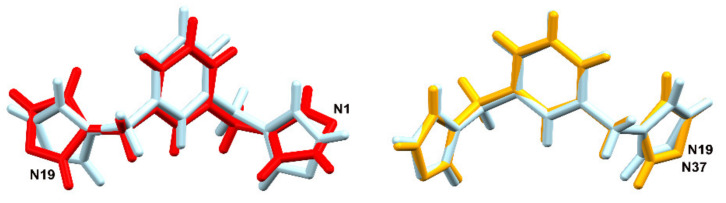
(**On the left**) overlay of the ligands containing N1 (red) and N19 (blue), (the RMS deviation after inversion is 0.9108 Å); and (**on the right**) overlay of the ligands containing N37 (orange) and N19 (blue), (the RMS deviation is 0.1832 Å) in **1**.

The ligand containing N1 shows a different orientation of one of the imidazole rings than the remaining two ligands (N19 and N37), the conformations of which do not differ much from one another (Figure 3).

**Figure 4:**
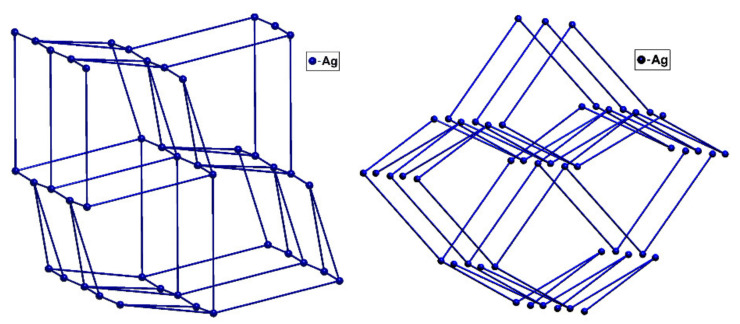
(**On the left**) representation of 5,5-c net of the topological type **5,5T188** disclosed in **1**; (**on the right**) 3-c uninodal net of the topological type **ths**.

Both silver ions show a distorted trigonal coordination environment with N-Ag-N angles ranging from 109.6(2)° to 132.5(2)°. Furthermore, there are argentophilic interactions present with a distance of 3.378 Å (van der Waals contact between the metal atoms is 3.44 Å) [20]. The 3D cationic network forms a 5,5-c net with point symbol: {3.5^5^.6^3^.7}{3^2^.5^4^.6^4^} and topological type 5,5T188 (Figure 4). A search in the TTO database indicates that this topological type has not been observed until now for any coordination compound [21]. A similar network can only be found in one purely organic compound, namely 3-hydroxy-3-isopropyl-4-trimethylsilylcyclopentanone (refcode: LAZNOP) [22], provided the hydrogen bonding is taken into account. A simplification which would not take into account the argentophilic interactions which are present would result in the formation of a more common **ths** topology with 3-c uninodal net and point symbol: {10^3^}, which up to now was observed in 457 cases for metal complexes, with examples such as catena-(bis(μ_3_-2,4,6-tris(pyridin-4-yl)-1,3,5-triazine)-hexachloro-tri-zinc nitrobenzene solvate) (refcode: IZUVUV) [23], catena-(bis(μ_3_-2,4,6-tris(4-pyridyl)triazine)-tris(di-iodo-zinc) perylene cyclohexane sesquikis(nitrobenzene) clathrate) (refcode: FARFUA) [24] and catena-[bis(μ_3_-2,4,6-tris(pyridin-4-yl)-1,3,5-triazine)-hexakis(iodo)-tri-zinc cyclohexane nobiletin solvate] (refcode: LABNEK) [25].

**Figure 5:**
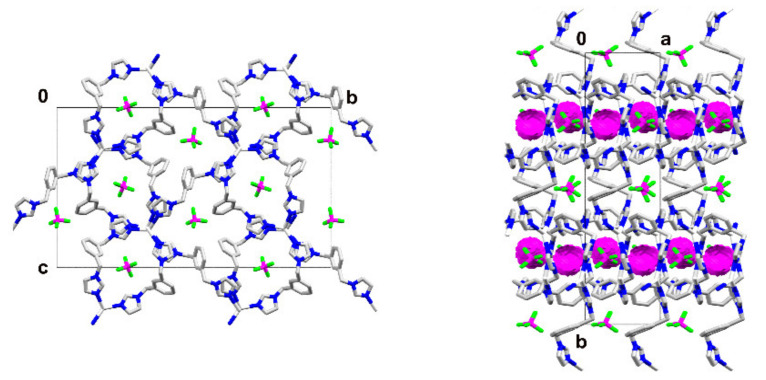
(**On the left**) packing diagram of **1** shown down the *a* axis; (**on the right**) packing diagram shown down the *c* axis exposing the voids (in pink) present between counterions; hydrogen atoms omitted for clarity.

The framework is stabilized by π-π interactions involving all imidazole rings with centroid to centroid distances ranging from 3.436(5) Å to 3.622(4) Å, as well as by C-H⋯π hydrogen bonds with the H atoms originating from the imidazole rings (N1, N37, N50) acting as donors and all three benzene rings acting as acceptors, with C⋯benzene ring centroid distances oscillating around 3.5 Å. Moreover, there is an extended net of C-H⋯F hydrogen bonds formed between the framework of the metal complex and the counterions, involving all F atoms. The counterions are occupying the channels formed along the *a* axis and the hydrogen bonds formed with the cationic framework are strong enough to keep the counterions in place, allowing for the formation of voids between adjacent ions with a calculated volume of 4363.0 Å^3^ per unit cell, accounting for 2.2% of the total cell volume (Figure 5; the Kitaigorodskii packing index is 69.8%) [26].

### 3.2. Crystal Structure of the Ag(I) Complex with PF_6_^−^ as Counterion ({[Ag(**bib**)]PF_6_}_n_, **2**)

**Figure 6:**
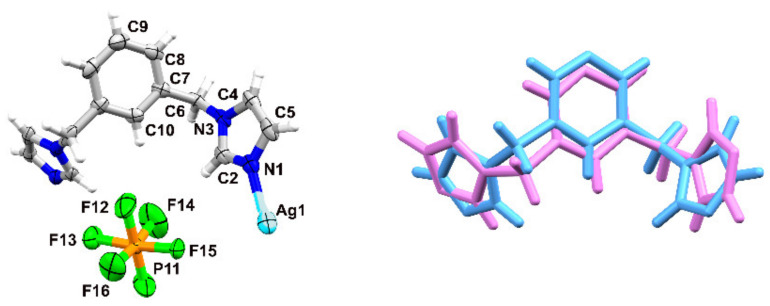
(**On the left**) a fragment of the helical chain in **2**, with the atomic displacement plot shown at 50% probability; unlabeled ligand atoms are generated by the symmetry operation *x*, ½ − *y*, 3/2 − *z;* for the counterion: 3/2 − *x*, 1 − *y*, *z*; (**on the right**) overlay of **2** (blue) with the ligand present in the related Ag(I) complex with **bimb** (magenta, methyl group removed for clarity); the RMS deviation is 1.0325 Å.

The compound crystallizes in the orthorombic space group *Pnna*, with half of the ligand as well as half of the silver cation and the counterion located on a two-fold axis in the asymmetric unit (Figure 6).

**Figure 7:**
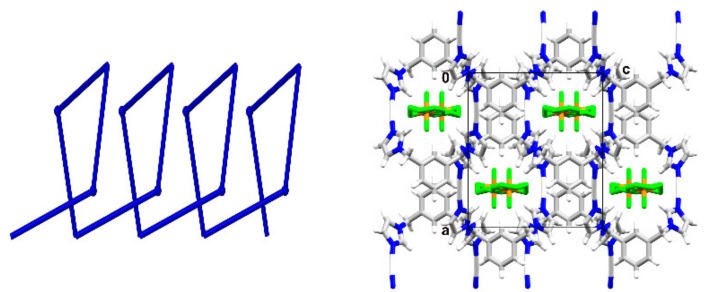
(**On the left**) representation of the helical chain formed by **2**; (**on the right**) packing diagram of **2** shown down the *b* axis.

The silver ions show a slightly deformed linear coordination with two N-atoms originating from two symmetry-related ligands in anti-conformation (symmetry operator: 3/2 − *x*, 1 − *y*, *z*) with an Ag-N distance of 2.079(5) Å and N-Ag-N angle of 176.1(2)°. The slight deviation from linearity might be the result of interactions of Ag(I) with F15 coming from the counterion (Ag···F = 3.037 Å). The two imidazole rings involved in the silver ion coordination environment are almost perpendicular to each other with an angle of 79.77° between their planes, compared to 35.20° for the corresponding compound with **bimb**. A comparison of the ligand conformations in these two compounds indicates among others a flip of one of the imidazole rings (Figure 6). Further comparison of the ligand conformation with the conformations adopted by the ligands in the Ag(I) complexes reported up to now shows the highest similarity with that (N19) in the crystal structure of **1** (RMS deviation of 0.3519 Å). The counterions template the formation of the helical chains (Figure 7) which are expanding along the *b* axis with a 10.589 Å pitch (in the case of the corresponding compound with **bimb** a waved chain was observed).

All F-atoms from the counterions are involved in interactions with the cationic complexes (Table 2), resulting in the formation of a 3D supramolecular assembly. This is further supported by weak π-π contacts formed by both N1 imidazole rings from two adjacent helices with a centroid–centroid distance of 3.760(3) Å.

### 3.3. Effect of Modifying the Ligand on the Resulting Crystal Structure

Comparing the crystal structures of Ag(I) complexes obtained under similar conditions by using ligands differing solely by the number of methyl substituents on the central benzene ring revealed that a higher number of methyl groups favors the formation of discrete molecules. The formation of a 3D polymeric network by combining AgBF_4_ with **bib** was unexpected, as the molar ratio does not reflect the initial reaction stochiometry. It shows once again that the results of the interplay of intermolecular interactions, leading to the most energetically favored form, might be hard if not impossible to predict. The discrepancy in unit cell parameters observed earlier for the isostructural compounds formed with AgBF_4_ and AgPF_6_ and **bimb** was already an indication that in this case the difference in volume of these counterions is significant and can lead to different products, which are not necessarily isostructural.

The contributions of the different forces stabilizing the crystal structures of the silver complexes with **bib**, **bimb** and **bitmb** and either the BF_4_^−^ or PF_6_^−^ counterion were estimated using Crystal Explorer [28].

**Figure 8:**
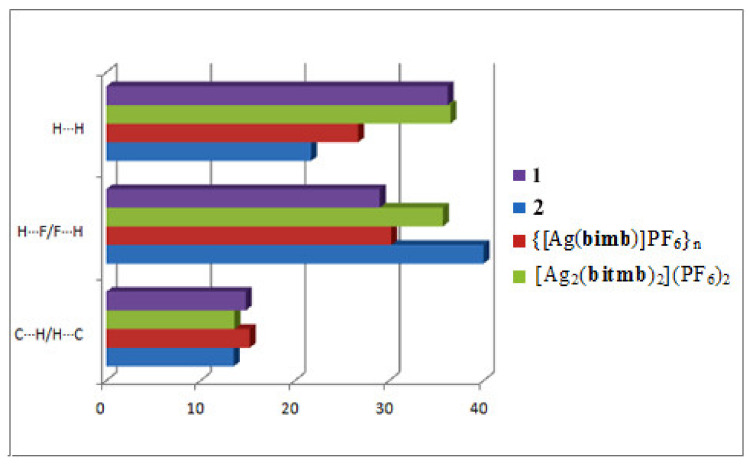
Estimated (%) contributions of selected intermolecular contacts to the Hirshfeld surface area for **1** and a series of Ag(I) complexes with PF_6_^−^.

As could be expected, C⋯H, F⋯H and H⋯H are essential forces contributing to the Hirshfeld surface areas of the compounds under investigation (Figure 8) with the input of the latter much higher in **1** than in **2**, in which F^…^H forces are dominant. The presence of voids in **1** and the higher contribution of hydrogen bonds in **2**, could be the reason for a slightly higher thermal stability of the latter complex, even though **1** shows higher dimensionality (the thermal decomposition starts at ca. 205 °C for **1** and 225 °C for **2**).

## 4. Conclusions

The final products of combining silver salts (BF_4_^−^, PF_6_^−^) with 1,3-bis(imidazol-1-ylmethyl)benzene obtained under the same reaction conditions (molar ratio L to M 1:1) are not isostructural, either with one another, or with the corresponding compounds obtained with 1,3-bis(imidazol-1-ylmethyl)-5-methylbenzene, 1,3-bis(imidazol-1-ylmethyl)-2,4,6-trimethylbenzene or with the silver complexes formed by 1,3-bis(imidazol-1-ylmethyl)benzene or 1,3-bis(imidazol-1-ylmethyl)-5-methylbenzene with CF_3_SO_3_^−^ as the counterion. Isostructurality in the case of the 1D Ag(I) complexes obtained with **bib**/**bimb** in the presence of triflate as the counterion was the result of the formation of 2D supramolecular layers and a rather loose packing. The formation of crystal structures with a much denser packing observed in the case of the complexes formed with **bimb** and counterions BF_4_^−^ and PF_6_^−^ as well as quite a discrepancy in their unit cell parameters, were already an indication that the corresponding compounds with **bib** might not be isostructural. This was confirmed by the present study. Moreover, the study revealed a unique topology among coordination compounds. Furthermore, it has also been shown that the absence of substituents or the presence of a single methyl group on the aromatic core of the ligand facilitate the formation of polymeric species with the ligand adopting an *anti*-conformation, whereas a higher number of substituents on the aromatic core of the ligand leads to the formation of dinuclear metallocycles with the ligand in *syn*-conformation under the same reaction conditions. This begs the question of to which extent the templating effect of counterions and ligand composition might be predictable. Hopefully, in silico methods might shed some light on this in the near future.

## Figures and Tables

**Table 1:** Crystal data and details of the refinement parameters for the crystal structures **1** and **2**.

Compound Reference	1	2
Chemical formula	C_42_H_42_Ag_2_B_2_F_8_N_12_	C_14_H_14_AgF_6_N_4_P
Formula mass	1104.23	491.13
Crystal system	Monoclinic	Orthorombic
*a*/Å	6.9785(8)	13.3957(19)
*b*/Å	32.556(4)	10.5887(15)
*c*/Å	19.241(2)	11.8827(17)
*α*/°	90	90
*β*/°	93.561(2)	90
*γ*/°	90	90
Unit cell volume/Å^3^	4363.0(9)	1685.5(4)
Temperature/K	100(2)	100(2)
Space group	*Cc*	*Pnna*
No. of formula units per unit cell, *Z*	4	4
Radiation type	Mo Kα	Mo Kα
Absorption coefficient, μ/mm^−1^	0.980	1.359
No. of reflections measured	13683	9153
No. of independent reflections	8270	1765
*R_int_*	0.0320	0.0438
Final *R*_1_ *^a^* values (*I* > 2*σ*(*I*))	0.0478	0.0562
Final *wR*_2_ *^b^* values (*I* > 2*σ*(*I*))	0.1112	0.1288
Final *R*_1_ *^a^* values (all data)	0.0513	0.0747
Final *wR*_2_ *^b^* values (all data)	0.1136	0.1395
Goodness of fit on *F*^2^	1.049	1.043

^a^*R*_1_ = ∑║*F*_o_| − |*F*_c_║/∑|*F*_o_|. ^b^ *wR*_2_ = {∑[*w*(*F*_o_^2^ − *F*_c_^2^)^2^]/∑[*w*(*F*_o_^2^)^2^]}^1/2^.

**Table 2:** Selected hydrogen bond parameters for **2**.

D-H⋯A	H⋯A/Å	D···A/Å	D-H···A/°
C5-H5⋯F12 ^i^	2.54	3.149 (7)	122
C6-H6A⋯F12 ^ii^	2.47	3.434 (7)	164
C8-H8⋯F13 ^iii^	2.36	3.280 (1)	162
C2-H2⋯F14	2.61	3.405 (16)	141
C4-H4⋯F15 ^iv^	2.68	3.444 (11)	137
C2-H2⋯F16 ^v^	2.31	3.150 (2)	147

Symmetry codes: ^i^ 1 − *x*, 1 − *y*, 1 − *z* ^ii^ *x*, 3/2 − *y*, 1/2 − *z* ^iii^ −1/2 + *x*, 3/2 − *y*,1/2 + *z* ^iv^ 1 − *x*, 1 − *y*, 1 − *z* ^v^ 3/2 − *x*, 1/2 + *y*, 1/2 − *z* [27].

## Data Availability

Data are contained within the article.
